# Wipe-Out Syndrome After PreserFlo MicroShunt Implantation in a Patient With Pseudoexfoliation Glaucoma

**DOI:** 10.7759/cureus.99396

**Published:** 2025-12-16

**Authors:** Jesspreet Kaur, Rupini Yogesvaran, Safinaz Mohd Khialdin, Norshamsiah Md Din

**Affiliations:** 1 Department of Ophthalmology, Hospital Canselor Tuanku Muhriz, Universiti Kebangsaan Malaysia, Kuala Lumpur, MYS

**Keywords:** minimally invasive glaucoma surgery (migs), preserflo microshunt, pseudoexfoliation glaucoma, uveitis, wipe-out syndrome

## Abstract

A 72-year-old male patient with multiple comorbidities was under our follow-up for bilateral advanced pseudoexfoliative glaucoma with uncontrolled intraocular pressure (IOP) despite maximum medical therapy. He had previously received panretinal photocoagulation laser in both eyes for ischemic central retinal vein occlusion and experienced one episode of bilateral anterior uveitis. The Humphrey visual field testing showed progressive worsening with macular split. Visual acuity was 6/9 in both eyes, with pale discs and a cup-to-disc ratio of 0.9. The patient underwent right eye PreserFlo MicroShunt implantation (InnFocus, Inc., a subsidiary of Santen Pharmaceutical Co., Ltd., Miami, FL, USA) with mitomycin C 0.02% in October 2022. Postoperatively, the course was complicated by hypotony with maculopathy. His vision slowly deteriorated from 6/18 to 6/24 and finally to perception of light within three weeks. Although there were no signs of overfiltration with a flat bleb, the IOP was unrecordable, and anterior chamber activity was one plus despite intensive topical steroids. A high-molecular-weight viscoelastic device was injected into the anterior chamber to increase the IOP to 15 mmHg, which spiked to 54 mmHg the following day. Anterior chamber paracentesis was performed twice, but his vision failed to improve and subsequently deteriorated to vague light perception.

Subconjunctival minimally invasive glaucoma surgery (MIGS) implantation, such as the PreserFlo MicroShunt, even in an eye with a previous single episode of uveitis, may still be complicated by ciliary shutdown and hypotony. The advanced stage of glaucomatous disc damage may tilt the course of the disease unfavorably, resulting in a "wipe-out" syndrome.

## Introduction

Glaucoma is an enduring ocular ailment that involves the optic nerve. If left untreated, glaucoma causes severe vision impairment and permanent blindness. Across the world, glaucoma continues to be a significant cause of blindness and the dominant cause of permanent vision loss [1].

Treatment for glaucoma is aimed at reducing the intraocular pressure (IOP) to prevent further disease progression. Over time, treatment options have expanded from topical and systemic medical therapy to laser treatments and various surgical procedures.

Minimally invasive glaucoma surgery (MIGS) is an expanding field that has gained much interest over the last decade, aiming to bridge the gap between medications and invasive surgery to reduce the IOP. These procedures aim to lower IOP effectively while minimizing disruption and postoperative complications [2]. The PreserFlo MicroShunt (InnFocus, Inc., a subsidiary of Santen Pharmaceutical Co., Ltd., Miami, FL, USA) is a bleb-forming MIGS device made of biocompatible and inert material with a 70 µm inner lumen size to maintain controlled aqueous flow and reduce the risk of both hypotony and obstruction by inflammatory debris [3]. The device's biocompatible composition and minimally invasive implantation approach help to minimize postoperative inflammation [4]. 

A meta-analysis found PreserFlo MicroShunt to be a beneficial addition to the treatment of moderate to severe disease with significant IOP reduction across various types of glaucoma and a good safety profile [4]. Reported complications include transient hypotony, hyphema, flat anterior chamber, high IOP, bleb leak, choroidal detachment, and anterior chamber cells or flare [3,5,6]. These issues were mostly treated conservatively or medically with a favorable outcome. Only a handful of sight-threatening adverse effects have been reported such as tube exposure [7], endophthalmitis after needling [8], hemorrhagic choroidal detachment [9], and malignant glaucoma [10]. Hence, a PreserFlo MicroShunt was implanted in this case. 

Wipe-out syndrome is characterized by an irreversible, sudden loss of central vision following filtering surgery, especially in advanced glaucoma patients with significant visual field loss [11]. Although the exact pathophysiology is not well understood, it has been associated with causes such as hypotony, choroidal detachment, and severe inflammation. While there have been reported cases of wipe-out syndrome in patients who underwent filtering surgery, no cases have been reported in those who had microinvasive glaucoma surgery [11,12].

We report a case of "wipe-out" syndrome following an uncomplicated augmented PreserFlo MicroShunt implantation that was complicated by prolonged hypotony from a combination of enhanced outflow and ciliary body dysfunction with reduced aqueous humour production.

This article was previously presented as an e-poster at the 39th Asia-Pacific Academy of Ophthalmology Congress 2024 on February 22-25, 2024. 

## Case presentation

A 72-year-old male patient was under our follow-up for pseudoexfoliation glaucoma. He had multiple systemic diseases, including diabetes mellitus, hypertension, dyslipidemia, and rheumatoid arthritis on a disease-modifying antirheumatic drug (DMARD). He had received complete panretinal photocoagulation laser in both eyes for ischemic central retinal vein occlusion 25 years ago and had one episode of bilateral anterior uveitis in 2020, which was successfully treated with topical dexamethasone eye drops.

**Figure 1:**
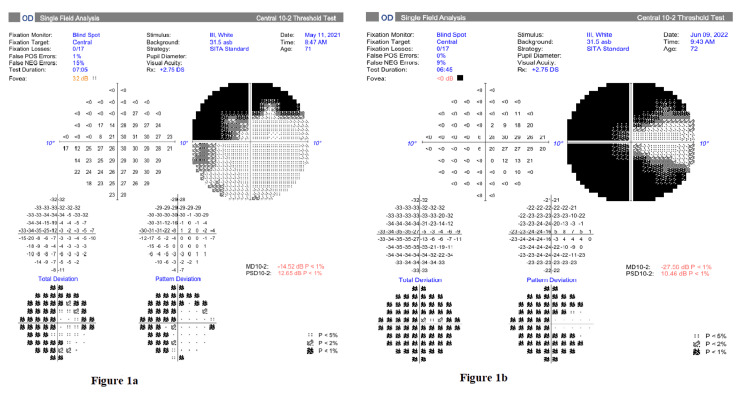
The right eye demonstrated a progressive deterioration in the 10-2 Humphrey visual field, with an MD worsening from -12.52 dB in 2021 (a) to -27.56 in 2022 (b), corresponding to progressive macular involvement. SITA: Swedish Interactive Thresholding Algorithm; MD: mean deviation; PSD: pattern standard deviation

The pseudoexfoliative glaucoma required maximum medical therapy over the years due to suboptimal IOP control. With IOP ranging from 14 mmHg to 22 mmHg, his visual field showed glaucomatous progression, more in his right eye with deterioration in mean deviation from -14.52 dB to -27.56 dB within a year, with macular split (Figure 1a, 1b). His vision was still good at 6/9 in both eyes with a pale disc and a 0.9 cup-to-disc ratio.

He underwent right eye PreserFlo MicroShunt implantation with mitomycin C 0.02% under sub-tenon anesthesia in October 2022. On day 1 postoperatively, his IOP was 6 mmHg with a flat bleb and a negative Seidel's test. His vision was 6/24, N48 with cellular activity of one plus. At this point, the optic disc was 0.9 pale, and there was no other evidence of macular striation and choroidal effusion to suggest hypotony. He was started on Gutt Dexamethasone 0.1% two hourly and Gutt Ciprofloxacin 0.3% two hourly.

**Figure 2:**
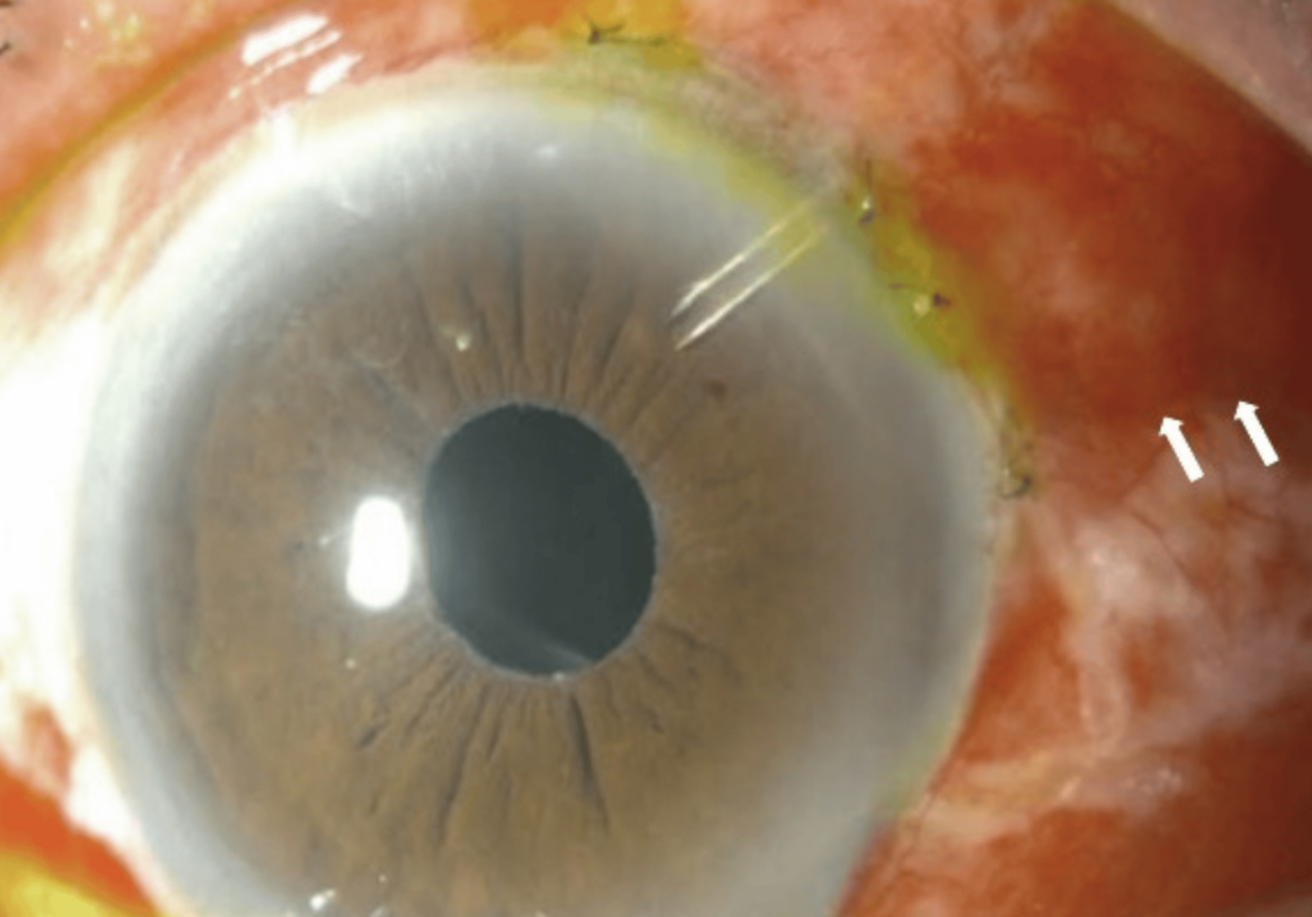
At postoperative week 1, the anterior chamber was deep, and bleb was shallow.

**Figure 3:**
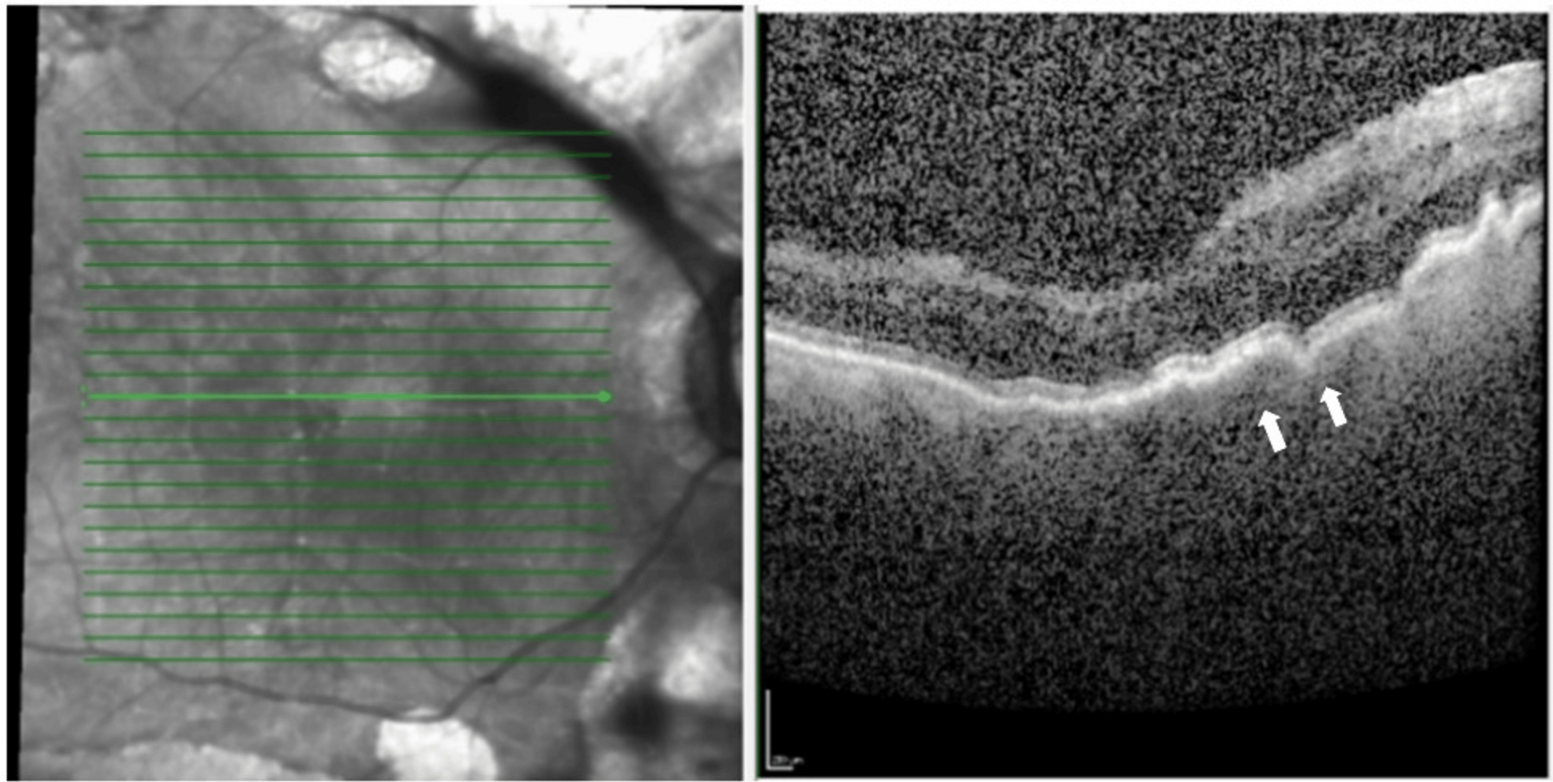
OCT of the macula showed the undulation of the retinal pigment epithelial layer at postoperative week 1. OCT: optical coherence tomography

Despite being compliant with his medications, at postoperative week 1 (Figure 2), we noticed that cellular activities had increased to two plus in a deep anterior chamber, IOP was 1, bleb was shallow, and macular striation was seen on posterior segment examination. Optical coherence tomography (OCT) of the macula showed the undulation of the retinal pigment epithelial layer (Figure 3), further confirming hypotony maculopathy. Intensive topical steroids (Gutt Prednisolone Acetate 1%) were given to treat the intense inflammation and ciliary shutdown.

At postoperative week 3, his vision had deteriorated to perception of light, unrecordable IOP, shallow anterior chamber depth, cellular activity of one plus, and no signs of wound leak or overfiltration. A high-molecular-weight viscoelastic device was injected into the anterior chamber for reformation, and IOP increased to 15 mmHg. The next day, his IOP shoot up to 54 mmHg. Anterior chamber paracentesis was performed to remove the retained viscoelastic device. However, his vision continued to be poor despite controlled IOP at 7-14 mmHg.

At postoperative week 10, his IOP began to increase despite subconjunctival 5-fluorouracil injection and limited bleb needling. IOP ranged between 17 mmHg and 40 mmHg. Currently, his vision remains vague light perception in the right eye with an IOP of 40 mmHg, without any pain and no glaucoma medication.

## Discussion

Sudden irreversible postoperative visual compromise in glaucoma patients with advanced disease, also known as "wipe-out" syndrome, is a devastating complication not only for the patient but also for their treating clinician. Literature reports commonly describe it following cataract surgery, glaucoma filtering surgery, or a combination of both [13-15], but none have been reported following PreserFlo MicroShunt surgery. Its incidence after glaucoma surgery is rather controversial, with reports ranging from as low as 0% [16] to as high as 13.6% [15].

Risk factors contributing to this irreversible visual loss comprise older age, advanced glaucoma characterized by preoperative macular split fixation on visual fields, the extent of quadrants involved, the presence of choroidal effusion, and previous episodes of retinal vein occlusion [13,15]. There have been numerous reports that patients with glaucomatous optic neuropathy have further nerve damage due to direct trauma, pressure, or ischemia following retrobulbar anesthesia or peribulbar anesthesia [17]. Francis et al. further proposed that postoperative extreme IOP fluctuations may be a risk factor for developing "wipe-out" syndrome [13].

Our patient was a 72-year-old man who had quite a number of these pre- and postoperative risk factors, including a history of retinal vein occlusion and an advanced stage of glaucoma with two-quadrant split fixation. During the early postoperative weeks, there was a period of hypotony which, after eliminating other causes, was attributed to ciliary body dysfunction or shutdown. Given the history of prior anterior uveitis and intense inflammation during the early postoperative period, we believe there was a marked reduction in aqueous humour production due to this dysfunction.

There have been a handful of cases that showed improvement in visual acuity following "wipe-out" syndrome [13,14]. Although not proven, these were attributed to the restoration of axoplasmic flow and improved optic nerve head perfusion, which may allow retinal ganglion cell function to recover once IOP remains stable [13]. Unfortunately, in our case, we did not achieve such success despite maintaining IOP within the target range for two months.

## Conclusions

MIGS devices like the PreserFlo MicroShunt, in an eye with a previous single episode of uveitis, may be complicated by a ciliary body dysfunction and reduced aqueous production. The advanced stage of glaucomatous disc damage may predispose the course of the disease to worsen when postoperative hypotony occurs, resulting in a "wipe-out" syndrome.
